# Dr. Elias L. Bissell

**Published:** 1905-11

**Authors:** 


### OBITUARY.

Dr. Elias L. Bissell, of Buffalo, died at his home of disease of the heart, November 1, 1905, aged 72 years. He had suffered ill health for several years but at last his death seemed sudden, as he did not appear worse than usual up to the last moment of his life.

Dr. Bissell was a native of Erie county, having been born in Lancaster in 1833, where he received his early education. He graduated in medicine from the University of Michigan in 1861 and was commissioned assistant surgeon of the 44th Infantry New York Voulunteers, October 11, 1861. He was promoted surgeon of the 22nd New York Infantry, November 20, 1862, and was mustered out with the regiment June 19, 1863, this being a two years' regiment. Upon his return he took up his residence at 2793 Main street, Buffalo, where he continued in medical practice until compelled to relinquish it by reason of ill health. He is survived by two daughters, both married and residing in Buffalo, and a brother, Anthony Bissell, of Lancaster. His wife died some years ago.

